# Altered Serotonin 1B Receptor Binding After Escitalopram for Depression Is Correlated With Treatment Effect

**DOI:** 10.1093/ijnp/pyae021

**Published:** 2024-05-02

**Authors:** M Gärde, G J Matheson, K Varnäs, P Svenningsson, E Hedman-Lagerlöf, J Lundberg, L Farde, M Tiger

**Affiliations:** Centre for Psychiatry Research, Department of Clinical Neuroscience, Karolinska Institutet and Stockholm Health Care Services, Region Stockholm, Stockholm, Sweden; Department of Psychiatry, Columbia University, New York, USA; Centre for Psychiatry Research, Department of Clinical Neuroscience, Karolinska Institutet and Stockholm Health Care Services, Region Stockholm, Stockholm, Sweden; Centre for Psychiatry Research, Department of Clinical Neuroscience, Karolinska Institutet and Stockholm Health Care Services, Region Stockholm, Stockholm, Sweden; Centre for Psychiatry Research, Department of Clinical Neuroscience, Karolinska Institutet and Stockholm Health Care Services, Region Stockholm, Stockholm, Sweden; Division of Psychology, Department of Clinical Neuroscience, Karolinska Institutet, Stockholm, Sweden; Centre for Psychiatry Research, Department of Clinical Neuroscience, Karolinska Institutet and Stockholm Health Care Services, Region Stockholm, Stockholm, Sweden; Centre for Psychiatry Research, Department of Clinical Neuroscience, Karolinska Institutet and Stockholm Health Care Services, Region Stockholm, Stockholm, Sweden; Centre for Psychiatry Research, Department of Clinical Neuroscience, Karolinska Institutet and Stockholm Health Care Services, Region Stockholm, Stockholm, Sweden

**Keywords:** Positron emission tomography, 5-HT_1B_ receptor, major depressive disorder, SSRI

## Abstract

**Background:**

Major depressive disorder (MDD) is commonly treated with selective serotonin reuptake inhibitors (SSRIs). SSRIs inhibit the serotonin transporter (5-HTT), but the downstream antidepressant mechanism of action of these drugs is poorly understood. The serotonin 1B (5-HT_1B_) receptor is functionally linked to 5-HTT and 5-HT_1B_ receptor binding and 5-HT_1B_ receptor mRNA is reduced in the raphe nuclei after SSRI administration in primates and rodents, respectively. The effect of SSRI treatment on 5-HT_1B_ receptor binding in patients with MDD has not been examined previously. This positron emission tomography (PET) study aimed to quantify brain 5-HT_1B_ receptor binding changes in vivo after SSRI treatment for MDD in relation to treatment effect.

**Methods:**

Eight unmedicated patients with moderate to severe MDD underwent PET with the 5-HT_1B_ receptor radioligand [^11^C]AZ10419369 before and after 3 to 4 weeks of treatment with the SSRI escitalopram 10 mg daily. Depression severity was assessed at time of PET and after 6 to 7 weeks of treatment with the Montgomery-Åsberg Depression Rating Scale.

**Results:**

We observed a significant reduction in [^11^C]AZ10419369 binding in a dorsal brainstem (DBS) region containing the median and dorsal raphe nuclei after escitalopram treatment (*P* = .036). Change in DBS [^11^C]AZ10419369 binding correlated with Montgomery-Åsberg Depression Rating Scale reduction after 3-4 (r = 0.78, *P* = .021) and 6-7 (r = 0.94, *P* < .001) weeks’ treatment.

**Conclusions:**

Our findings align with the previously reported reduction of 5-HT_1B_ receptor binding in the raphe nuclei after SSRI administration and support future studies testing change in DBS 5-HT_1B_ receptor binding as an SSRI treatment response marker.

**Significance Statement**

Despite being widely employed in the treatment of major depressive disorder (MDD) the mechanism of action of selective serotonin reuptake inhibitors (SSRIs) is not fully understood. The serotonin 1B (5-HT_1B_) receptor has been proposed as a potential target for antidepressants based on animal models of MDD, MDD case-control studies, and the effects of drugs that bind to the 5-HT_1B_ receptor. In this positron emission tomography study, we show that 5-HT_1B_ receptor binding is reduced in the dorsal brainstem (DBS) in MDD patients after 3 to 4 weeks of SSRI treatment. DBS 5-HT_1B_ receptor binding change was strongly correlated to reduction in depression severity. To our knowledge, a significant correlation between change in 5-HT_1B_ receptor binding and change in depression severity has not been reported previously. These results contribute to our understanding of the neural mechanisms underlying SSRI treatment of depression and could have implications for improving the efficacy of antidepressant therapies.

## INTRODUCTION

Major depressive disorder (MDD) is a common (Kessler et al., 2003) and debilitating brain disorder ranking among the top contributors to global disability (World Health Organization, 2017; James et al., 2018). First-line MDD treatment is generally a selective serotonin reuptake inhibitor (SSRI), to which roughly one-half of patients respond (Rush et al., 2006). However, the mechanism of action of SSRIs is still not fully understood, and there are currently no clinically available biological markers that predict SSRI response.

Although SSRIs inhibit the serotonin transporter (5-HTT) in the brain within hours (Lundberg et al., 2012), a clinically noticeable antidepressant effect generally requires weeks to manifest. This delayed response indicates that improved mood is the result of downstream adaptive events rather than 5-HTT inhibition per se. For instance, the serotonin 1 (5-HT_1_) receptor family has been proposed to be involved in the antidepressant mechanism of SSRIs based on desensitization of 5-HT_1_ receptors at time points corresponding to the onset of antidepressant effect in humans (Bel and Artigas, 1993; Piñeyro and Blier, 1996; Newman et al., 2004; Nautiyal and Hen, 2017). While the majority of research on serotonin 5-HT_1_ receptors has focused on the serotonin 1A (5-HT_1A_) receptor, in recent years, the serotonin 1B (5-HT_1B_) receptor has been put forward as a potential target for antidepressant treatment (Tiger et al., 2018). This hypothesis has received support from studies of animal models of depression, MDD case-control studies, and reports on antidepressive like effects of 5-HT_1B_ receptor agonists and antagonists (Ruf and Bhagwagar, 2009; Zhang et al., 2011; Tiger et al., 2016, 2018).

5-HT_1B_ receptors are expressed on serotonergic neurons where they act as autoreceptors (Barnes and Sharp, 1999), reducing synaptic 5-HT release through negative feedback mechanisms (Middlemiss and Hutson, 1990; Bühlen et al., 1996; Roberts et al., 1997; Barnes and Sharp, 1999). In addition, 5-HT_1B_ receptors also function as heteroreceptors regulating the release of other neurotransmitters, including acetylcholine, GABA, and dopamine (Barnes and Sharp, 1999; Sari, 2004). Autoradiography studies have revealed high 5-HT_1B_ receptor densities in the basal ganglia and intermediate densities in cortical areas and in the raphe nuclei (Varnäs et al., 2001, 2004). Given the limited effect on 5-HT_1B_ receptor binding after lesioning of serotonergic neurons or selective knockdown of 5-HT_1B_ autoreceptors (Pranzatelli et al., 1996; Nautiyal et al., 2016), it appears likely that heteroreceptors constitute a substantial component of the 5-HT_1B_ receptor signal in the brain. The median and dorsal raphe nucleus (MRN and DRN) together contain the majority of the serotonergic cell bodies of the CNS (Hornung, 2003). The MRN projects to the hippocampus, limbic structures, and the nucleus accumbens and has been implicated in the pathophysiology of depression (Lechin et al., 2006) and reward-seeking behavior (Szumlinski et al., 2004; Kawai et al., 2022). Furthermore, the MRN region has been put forward as important to SSRI response in MDD patients (Deakin, 2003; Lanzenberger et al., 2012) and reduced 5-HT_1B_ receptor binding after cognitive behavioral therapy has been reported in a dorsal brain stem region encompassing the MRN (Tiger et al., 2014).

The augmentation of 5-HTT activity subsequent to the activation of 5-HT_1B_ receptors, and conversely, the diminished reuptake of 5-HT following the administration of a 5-HT_1B_ receptor antagonist (Daws et al., 2000; Hagan et al., 2012; Montañez et al., 2014), implies a reciprocal relationship wherein 5-HT_1B_ receptors potentially influence synaptic 5-HT levels through interactions with 5-HTT. The functional interplay between these two serotonin regulating proteins is anatomically supported by the correlation between 5-HT_1B_ receptor binding and 5-HTT binding in hippocampus and cortical regions in healthy volunteers (Svensson et al., 2022). Moreover, experimental evidence suggests an important role of 5-HT_1B_ receptors in the delayed behavioral responses to SSRIs. Knockout studies demonstrate that 5-HT_1B_ receptors limit the 5-HT elevating effect of SSRIs (Knobelman et al., 2001; Malagié et al., 2001; Nautiyal et al., 2016). During prolonged exposure to SSRIs, reduced sensitivity to 5-HT_1B_ receptor agonists and reduced raphe nuclei 5-HT_1B_ receptor mRNA has been observed in rats at time points corresponding to the onset of antidepressant effects in humans (Neumaier et al., 1996; Anthony et al., 2000; Lifschytz et al., 2004; Newman et al., 2004). Finally, the absence of an anti-immobility effect of SSRIs in 5-HT_1B_ receptor knockout mice during the forced swimming test implicates a potential role for the 5-HT_1B_ receptor in the antidepressant mechanism of action of SSRIs (Trillat et al., 1998).

Positron emission tomography (PET) and the selective radioligand [^11^C]AZ10419369 enables quantification of 5-HT_1B_ receptor binding in vivo (Pierson et al., 2008; Varnäs et al., 2011). A previous PET-study has demonstrated a reduction in 5-HT_1B_ receptor binding in the raphe nuclei in nonhuman primates after a clinically relevant dose of citalopram (Yang et al., 2019). Similarly, numerically reduced 5-HT_1B_ receptor binding in the raphe nuclei together with significantly increased binding in cortical areas was observed in human volunteers given a clinically relevant dose of escitalopram (Nord et al., 2013). Whether 5-HT_1B_ receptor changes also occur after SSRI treatment in MDD patients, and to what degree such changes are related to treatment effects, have not been studied previously.

In this clinical PET study, we hypothesized that SSRI treatment of patients with MDD would induce a decrease in 5-HT_1B_ receptor binding in the dorsal brainstem and an increase in serotonin projection areas. We also hypothesized that 5-HT_1B_ receptor change would be related to reduction in depression severity.

## MATERIALS AND METHODS

Study aims, experimental design, and analysis methods were preregistered at aspredicted.org on January 6, 2020, before any data collection. Preregistration study number #33267, available at https://aspredicted.org/i44fh.pdf.

### Sample

All participants provided oral and written consent before initiation of any study-related event. The study was approved by the Swedish Ethical Review Authority in Stockholm and the Swedish Medical Products Agency in Uppsala and was performed in accordance with the Declaration of Helsinki and International Conference on Harmonization/Good Clinical Practice guidelines.

### Participants

Study participants were recruited either via Gustavsberg University Primary Care Center or through online advertising. The diagnosis of MDD was confirmed by a psychiatrist (M.G. or M.T.) at a face-to-face visit using the Mini-International Neuropsychiatric Interview (Sheehan et al., 1998). Inclusion criteria were ongoing MDD episode with a Montgomery-Åsberg Depression Rating Scale (MADRS) (Montgomery and Asberg, 1979) of >20, indicating at least moderate depression severity and otherwise healthy according to medical history, physical exam, blood tests, and urine test. Exclusion criteria were ongoing antidepressant treatment or any medication with effect on serotonergic transmission, bipolar disorder, psychotic disorder, substance use disorder, organic CNS disorder, pregnancy, hypersensitivity to escitalopram, claustrophobia, or otherwise unsuitable for MRI. Minimum washout time for drugs with serotonergic effect was 2 months. Table 1 outlines the characteristics of the eight patients that completed the study according to protocol. One MDD patient had previously been diagnosed with attention deficit disorder and another with generalized anxiety disorder. No other psychiatric co-morbidities were present in the study sample.

**Table 1.** Demographics and study variables

Variable	PET1	PET2	At 6–7 wk of treatment	*P* ^*a*^
Age	38.4 ± 7.58			
Sex	4M/4F			
Injected radioactivity (MBq)	330 ± 25.3	361 ± 43.3		.212
Specific radioactivity (GBq/µmol)	418 ± 130	407 ± 161		.865
Injected mass (µg)	0.45 ± 0.25	0.49 ± 0.21		.426
MADRS	25.5 ± 2.28	14.00 ± 9.24	7.75 ± 5.25	.009, <.001^*b*^

Abbreviations: MADRS, Montgomery–Åsberg Depression Rating Scale; PET, positron emission tomography.

^*a*^Paired 2-tailed *t* tests.

^*b*^Refers to MADRS change from PET 1 to PET 2 and clinical follow up respectively.

### Study Design

MDD patients commenced treatment with escitalopram 10 mg daily the day after their first PET examination (PET 1). A follow-up PET examination (PET 2) was performed after 3 to 4 weeks (mean ± SD = 3.5 ± 0.5) of treatment. To prevent potential confounding effects from diurnal variation in serotonergic function, all patients underwent follow-up PET at the same time of day as their baseline examination. Depression severity was assessed using MADRS at PET 1 and PET 2 and at clinical follow-up 6 to 7 weeks after start of escitalopram treatment. The serum escitalopram concentration at PET 2 was measured in 4 samples obtained at the following time points: one hour before the PET examination, at radioligand injection, 45 minutes into examination, and at the end of PET.

### PET- Procedures

To minimize head movement, a plaster based head fixation device was custom made for each patient (Bergström et al., 1981). All patients were examined using a High-Resolution Research Tomograph (HRRT) scanner (Siemens Molecular Imaging, Knoxville, TN, USA) with a maximum spatial resolution of approximately 1.5 mm full-width-half-maximum (Varrone et al., 2009). To account for signal attenuation, a transmission scan was performed before each PET (Varrone et al., 2009). The radioligand [^11^C]AZ10419369 was prepared as previously described (Pierson et al., 2008), diluted in 10 mL of saline and injected into the antecubital vein followed by a 10-mL saline bolus. Injected radioligand characteristics are described in detail in Table 1. Dynamic list-mode PET data were acquired for 93 minutes and reconstructed into 37 consecutive frames (8 × 10-second, 5 × 20-second, 4 × 30-second, 4 × 60-second, 4 × 180-second, and 12 × 360-second frames).

### MRI Procedures

T1-weighted MRI images were acquired using a 3T GE Signa system Discovery MR750 (GE Medical Systems, USA). The T1 sequence was a 3D EFGRE BRAVO protocol with repetition time = 8.15 milliseconds, echo time = 3.18 milliseconds, matrix = 256 × 256 × 176 and slice thickness = 1 mm.

### Image Acquisition and Analysis

PET images were reconstructed and motion corrected using a rigid-body registration (Schain et al., 2013). T1-weighted MRI images were co-registered to time-weighted summated PET-images using Statistical Parametric Mapping version 12 (SPM12, Wellcome Department of Cognitive Neurology, University College, London, UK). Four regions of interest (ROIs) were selected based on their relevance for MDD pathophysiology according to the literature (Murrough et al., 2011; Savitz and Drevets, 2013; Tiger et al., 2016, 2020): the anterior cingulate cortex (ACC), the orbitofrontal cortex (OFC), the hippocampus, and a dorsal brainstem (DBS) region containing the dorsal and median raphe nuclei. The Freesurfer analysis suite (version 6.0, http://surfer.nmr.mgh.harvard.edu) was applied on individual MRI data to delineate the ACC, OFC, and hippocampus ROIs. Bilateral ROIs were merged; for example, the left and right ACC was combined into one ACC ROI. In a template-based method, recently developed by our group, a combined MRN, DRN, and periaqueductal gray ROI has been shown to display superior test-retest metrics compared with manual delineation (Veldman et al., 2022). However, for individual raphe nuclei, the minimal detectable difference was similar for manual and template ROIs. In accordance with our preregistration, the DBS ROI was defined manually in a manner previously described (Kranz et al., 2012; Tiger et al., 2014) to include primarily the MRN with marginal contribution from the caudal DRN.

Using the MRI-based co-registration matrix, regional time activity curves were obtained by projecting ROIs onto the realigned dynamic PET image. For quantification, we employed the Simplified Reference Tissue Model (SRTM) using the cerebellar grey matter as a reference region due to previous autoradiography findings of negligible 5-HT_1B_ receptors in this brain region (Varnäs et al., 2004). The cerebellum ROI was defined using Freesurfer-derived cerebellar gray matter trimmed in an automated process (Svensson et al., 2019) to reduce the risk of spillover of radioactivity from adjacent structures. Owing to the small size of the DBS ROI and its relatively low binding, the time activity curves had a possible high degree of measurement error, which can give rise to inaccurate *BP*_ND_ estimates using SRTM. In accordance with our preregistration, DBS *BP*_ND_ was for this reason estimated using the wavelet-aided parametric imaging approach (Cselényi et al., 2006; Schain et al., 2013), which has previously been shown to reduce measurement variability compared with SRTM for estimation of 5-HT_1B_ raphe nuclei *BP*_ND_ (Nord et al., 2014). This method employs the non-invasive Logan plot fitted with a multilinear regression to estimate *BP*_ND_, for which we defined a t* value of 26 minutes. The regional *BP*_ND_ estimates were obtained by applying each individual’s DBS mask to their parametric image and calculating the mean *BP*_ND_ value within that mask.

### Statistics

The preregistered statistical analysis for this study can be found at: https://aspredicted.org/i44fh.pdf. Here, we specified that we would estimate treatment effects using a linear mixed effects model applied to log-transformed *BP*_ND_ values. *BP*_ND_ values were log-transformed to account for the expected similar proportional variance within regions, as well as the expected proportional effects of treatment. Our a priori hypotheses, defined in the preregistration, were that we would find increased *BP*_ND_ following treatment in the serotonin projection regions and decreased *BP*_ND_ in the dorsal brainstem. The model was to be defined with a random intercept for individual and fixed effects of brain region and treatment along with an interaction effect between region and treatment.

The relationship between change in symptom scores, measured before and after treatment using MADRS, and change in *BP*_ND_ values was assessed using Pearson correlation coefficients.

Statistical analysis and data visualization were performed using R (version 4.2.1 “Funny-looking Kid”) (R Core Team, 2022).

### Deviations From the Preregistration Protocol

In our preregistration, we specified that 20 patients would be included in the study. Owing to the COVID pandemic, as well as the retirement of the PET system on which the data were collected, we were unable to reach this target.

Upon examination of the *BP*_ND_ values, we became aware that the log transformation was inappropriate for the DBS and hippocampus regions because of their low mean. Although biological treatment effects are expected to be approximately proportional to the mean, errors in estimates of *BP*_ND_ are not. Because of the effect of the logarithmic transform to stretch values in the low range, this transformation results in an exaggeration of the measurement error in these regions, which in turn gives rise to unplausible treatment effects. For instance, 2 individuals exhibited Δ*BP*_ND_ values of less than 0.5 in the original scale within the DBS, yet when transformed to Δlog(*BP*_ND_) to examine proportional changes, pretreatment binding was over 500% their posttreatment binding. Given our a priori hypothesis of regionally specific changes, the excessive proportional variation was deemed problematic, especially considering our relatively small sample size. For this reason, we did not make further use of the log-transformation. Without log-transformation, mixed effects models across regions with different mean binding are not appropriate owing to the differences in variance, which would give rise to heteroscedasticity. Instead, to examine longitudinal changes, we made use of the alternative modelling strategy defined in the preregistration, namely of using paired sample *t* tests applied to untransformed *BP*_ND_ values for each of the individual ROIs. In each case, we tested for changes in the directions hypothesized in our preregistration.

## RESULTS

Ten patients met all the inclusion and none of the exclusion criteria for the study. One of the included patients withdrew consent before study initiation, and another patient was excluded due to claustrophobic anxiety during PET 1, precluding the examination. This left eight patients who completed the study according to protocol.

The four individual escitalopram serum concentrations obtained in association with PET 2 ranged between 29 and 94 nmol/L (mean ± SD = 52.5 ± 18.2) in line with earlier observations from clinical samples (Reis et al., 2007). There were no significant differences in injected radioactivity, molar radioactivity, or injected mass between PET 1 and PET 2. All patients maintained their prescribed dose of 10 mg escitalopram throughout the study. Six out of eight patients (75%) responded to treatment, defined as a >50% reduction in MADRS score at the 6- to 7-week clinical evaluation (mean ∆MADRS ± SD = −17.6 ± 4.9).

**Figure 2.**
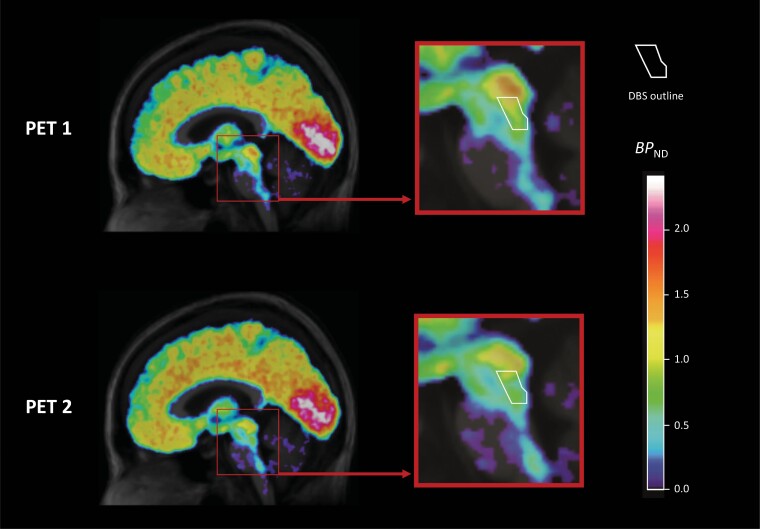
Parametric positron emission tomography (PET) image of [^11^C]AZ10419369 *BP*_ND_ before (PET 1) and after (PET 2) treatment with escitalopram 10mg. Parametric images of averaged [^11^C]AZ10419369 binding at PET 1 and PET 2 normalized to MNI space. Closeup of brainstem area, with an outline of the summed dorsal brainstem (DBS) region of interest (ROI) for the eight participants normalized to MNI space. Normalization was performed using SPM12.

As we predicted in our preregistration, paired *t* tests revealed a significant reduction of [^11^C]AZ10419369 binding (*BP*_ND_) in the DBS ROI (*P* = .036) at PET 2 corresponding to (mean ± SD) 24.4 ± 3.2 days of treatment with escitalopram. (Table 2, Figure 2). Contrary to our preregistered hypothesis, there were no significant increases in any of the cortical projection regions: ACC (*P* = .391), OFC (*P* = .362), and hippocampus (*P* = .738) (Table 2). A plot of longitudinal [^11^C]AZ10419369 *BP*_ND_ for each ROI is available in the supplement.

**Table 2.** Regional [^11^C]AZ10419369 *BP*_ND_ before (PET1) and after 3 weeks of escitalopram 10mg daily (PET2)

Region of interest	[^11^C]AZ10419369 *BP*_ND_		% *BP*_ND_ change	*P* ^*a*^
	PET 1	PET 2		
Anterior cingulate cortex	1.16 ± 0.18	1.19 ± 0.29	2.9	.391
Orbitofrontal cortex	1.17 ± 0.19	1.20 ± 0.26	2.8	.362
Hippocampus	0.38 ± 0.14	0.36 ± 0.18	−6.8	.738
Dorsal brainstem	0.71 ± 0.37	0.57 ± 0.33	−19.6	.036

Abbreviations: PET, positron emission tomography.

^*a*^Paired 1-sided *t* test.

**Figure 1.**
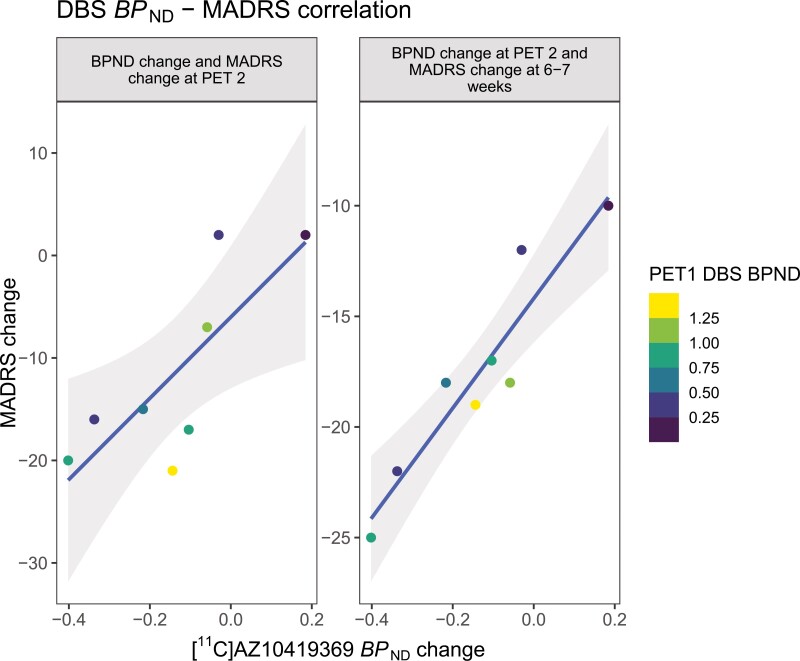
DBS *BP*_ND_–MADRS correlation. Scatterplots of change in MADRS to change in DBS [^11^C]AZ10419369 *BP*_ND_ after treatment with escitalopram 10 mg at PET 2 and at 6 to 7 weeks. Regression line in blue with shaded 95% CI. Color represents dorsal brainstem (DBS) [^11^C]AZ10419369 *BP*_ND_ at PET 1. MADRS, Montgomery-Åsberg Depression Rating Scale.

The *BP*_ND_ changes in the DBS region significantly correlated with the antidepressive effect rated with MADRS at PET 2 (r = 0.78, *P* = .021) and at 6 to 7seven weeks of treatment (r = 0.94, *P* <.001; Figure 1). There were no significant correlations between *BP*_ND_ change in projection regions and change in MADRS scores.

## DISCUSSION

In this clinical PET study of the molecular mechanism of action of SSRIs, we observed reduced [^11^C]AZ10419369 *BP*_ND_ in the DBS of MDD patients after 3 to 4 weeks of treatment with escitalopram. The *BP*_ND_ changes correlated with the antidepressant effect rated with MADRS at PET 2 and at the follow-up clinical evaluation after 6 to 7 weeks of treatment. The DBS ROI encompass especially the median raphe nucleus, housing a substantial proportion of CNS serotonergic cell bodies (Hornung, 2003), with projections to brain regions implicated in mood disorders (Vertes et al., 1999; Hornung, 2003).

The reduced [^11^C]AZ10419369 *BP*_ND_ in the DBS could represent either a reduction in 5-HT_1B_ receptor density (B_max_) or an increase in synaptic 5-HT. The latter interpretation must be considered given the [^11^C]AZ10419369 displacement previously reported after acute administration of the 5-HT releasing compound fenfluramine as well as supratherapeutic doses of SSRIs (Udo de Haes et al., 2006; Finnema et al., 2010; Nord et al., 2013). However, in a previous study performed in our group, a clinical dose of escitalopram did not significantly displace [^11^C]AZ10419369 binding in the brain in healthy volunteers (Nord et al., 2013). In addition, we observed no correlation between [^11^C]AZ10419369 and concentrations of 5-HT and its metabolite 5-HIAA in cerebrospinal fluid of healthy volunteers (Tiger et al., 2016). On the other hand, receptor downregulation as a response to receptor activation is an adaptation known to occur in a range of membrane receptor types (Thomas and Hoffman, 1986; Hadcock and Malbon, 1988). SSRIs increase synaptic 5-HT availability, and reduction in 5-HT_1B_ receptor density has been observed in vitro after administration of 5-HT and 5-HT_1B_ receptor agonists (Pleus and Bylund, 1992; Unsworth and Molinoff, 1992). Additionally, a raphe-confined 5-HT_1B_ receptor mRNA reduction after chronic SSRI administration is a replicated finding in experimental animals and observed at different time points (Neumaier et al., 1996; Anthony et al., 2000). An SSRI-induced downregulation of raphe 5-HT_1B_ receptors is also supported by the desensitization of 5-HT_1B_ receptors observed in rats after chronic exposure to SSRIs (Newman et al., 2004). To conclude, although an increase in synaptic 5-HT cannot be ruled out, we believe that the reduced [^11^C]AZ10419369 DBS *BP*_ND_ most likely represents a reduction in DBS 5-HT_1B_ receptor density.

The other major finding in the present study is the correlation between reduction in MADRS score and reduced DBS 5-HT_1B_ receptor binding. An important role of 5-HT_1B_ receptors in the behavioral effects of SSRIs is underscored by the absence of an anti-immobility effect of SSRIs in the forced swim test of 5-HT_1B_ receptor knockout mice (Trillat et al., 1998). However, the behavioral effects of concurrent SSRI and 5-HT_1B_ receptor antagonist administration in animal models of depression has so far been mixed, with reports both of enhanced (Tatarczyńska et al., 2004), and decreased (O’Neill et al., 1996), SSRI response. Decreased 5-HT_1_ receptor attenuation of the 5-HT elevating effects of SSRIs has been postulated as a key component in the delayed therapeutic effects of these drugs (Carhart-Harris and Nutt, 2017; Nautiyal and Hen, 2017). In humans, this has been tested primarily for the 5-HT_1A_ receptor, where SSRI treatment for MDD indeed has been associated with a reduction of raphe 5-HT_1A_ receptor binding, although this change did not correlate with treatment response (Sargent et al., 2000; Gray et al., 2013). To our knowledge, the strong correlation between MDD symptom alleviation and reduced binding after SSRI is unique for the 5-HT_1B_ receptor. The MADRS scale has been developed to be sensitive to changes in symptoms and signs after pharmacological treatment for depression (Montgomery and Asberg, 1979). Two out of four of the antidepressants that MADRS is based on, clomipramine and amitriptyline, share 5-HTT inhibition as part of their mechanism of action (Lundberg et al., 2012), which adds to the validity of the correlation between reduction of 5-HT_1B_ receptor binding and MADRS after escitalopram for depression.

Increases in frontal cortex 5-HT_1B_ receptor binding has been reported after single doses of escitalopram (Nord et al., 2013; Yang et al., 2019), and a trend toward an increase in frontal cortex and hippocampal 5-HT_1B_ receptor mRNA has been observed in rats after 1 week of fluoxetine exposure (Neumaier et al., 1996). However, in longer time series, no 5-HT_1B_ receptor mRNA changes outside the raphe nuclei has been detected (Neumaier et al., 1996; Anthony et al., 2000), indicating that 5-HT_1B_ receptor increases in serotonin projection areas after SSRI exposure may be transient. In the present study, no significant SSRI 5-HT_1B_ receptor effect was observed outside the DBS. Given our small sample size, the risk of a type II error must be considered, and a small to moderate SSRI effect in projection areas should not be ruled out. However, a localized raphe-specific effect of chronic SSRIs appears to be the case for the 5-HT_1A_ receptor, with changes in binding exclusively in the raphe nuclei in MDD patients after 6 weeks of SSRI treatment (Gray et al., 2013).

An important limitation of this study is the lack of a treatment comparator. We thus cannot rule out that 5-HT_1B_ receptor changes were driven by effects unrelated to escitalopram exposure. For example, high PET 1 DBS *BP*_ND_ could lead to reduced *BP*_ND_ at PET 2 simply through regression towards the mean. However, this scenario appears unlikely given the lack of correlation between PET 1 DBS *BP*_ND_ and DBS *BP*_ND_ change at PET 2 (r = −0.42, *P* = .304; Figure 1). Another possibility is that the reduction in 5-HT_1B_ receptor binding is a feature of reduced depression rather than a result of 5-HTT inhibition by escitalopram. Future PET studies of 5-HT_1B_ receptor binding in patients with MDD treated with a nonserotonergic antidepressant is needed to address this research question. The present response rate of 75% is higher than that seen in clinical samples, which might be attributed to a comparably strong placebo effect. However, response rates may also be related to the careful selection of study participants, of whom most lacked psychiatric co-morbidities. In this small study, with a limited number of preregistered research questions, the risk of a type II error was deemed higher than that of falsely rejecting the null hypothesis. We therefore opted against explicit correction for multiple comparisons. Finally, a limitation with the applied methodology is that PET cannot distinguish between auto- and heteroreceptors, meaning that the reduction in DBS [^11^C]AZ10419369 *BP*_ND_ could pertain to 5-HT_1B_ receptors located on either serotonergic or nonserotonergic neurons, or both.

In conclusion, escitalopram treatment reduced 5-HT_1B_ receptor binding in the dorsal brainstem in MDD patients corroborating preclinical findings of a time-dependent SSRI effect in this brain region. The significant correlation between DBS [^11^C]AZ10419369 *BP*_ND_ change and MADRS score reduction provides support for the involvement of 5-HT_1B_ receptors in the antidepressant mechanism of action of escitalopram. Randomized controlled trials are needed to test change in raphe 5-HT_1B_ receptor binding as an SSRI treatment response marker.

## Supplementary Material

pyae021_suppl_Supplementary_Materials

## Data Availability

The data supporting the findings of this study are available on request. The data are not publicly available due to ethical and privacy restrictions.
