# Supplementary material for: Altered Serotonin 1B Receptor Binding After Escitalopram for Depression Is Correlated With Treatment Effect
Source: Int J Neuropsychopharmacol. 2024 May 2;27(5):pyae021. doi: 10.1093/ijnp/pyae021 (PMC11119883; doi:10.1093/ijnp/pyae021)
Supplement: pyae021_suppl_Supplementary_Materials [file pyae021_suppl_supplementary_materials.doc]

Altered serotonin 1B receptor binding after escitalopram for depression is correlated with treatment effect

**Supplementary material**

**
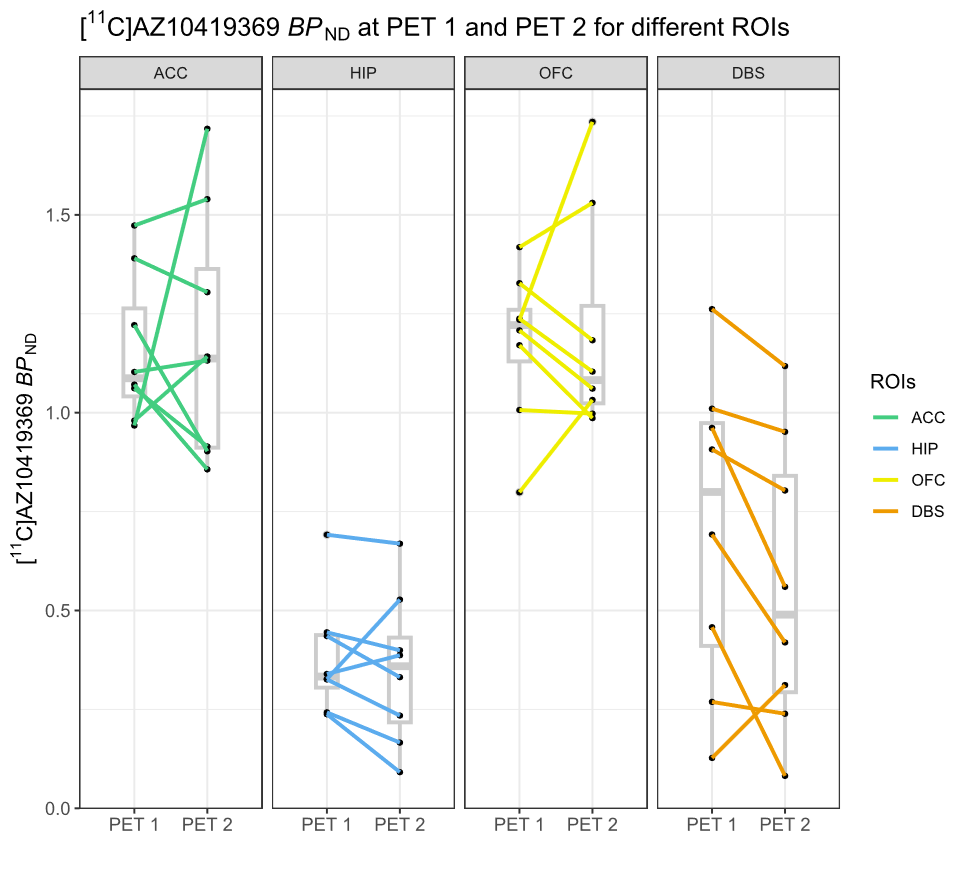
**

**Supplementary figure 1**:

Boxplots and lineplots of [^11^C]AZ10419369 *BP*_ND_ in different brain regions at PET1 and PET2. ACC, anterior cingulate cortex; HIP, hippocampus; OFC, orbitofrontal cortex; DBS, dorsal brainstem
